# Daily physical activity in older adults across levels of care: the HUNT Trondheim 70 + study 

**DOI:** 10.1186/s11556-024-00355-6

**Published:** 2024-07-17

**Authors:** Astrid Ustad, Karen Sverdrup, Gro Gujord Tangen, Øystein Døhl, Beatrix Vereijken, Pernille Thingstad, Nina Skjæret-Maroni

**Affiliations:** 1 Department of Neuromedicine and Movement Science, Faculty of Medicine and Health Science, Norwegian University of Science and Technology, Edvard Griegs Gate 8, 7030 Trondheim, Norway; 2 Norwegian National Centre for Ageing and Health, Vestfold Hospital Trust, Oslo, Norway; 3 Department of Geriatric Medicine, Oslo University Hospital, Oslo, Norway; 4 Oslo Metropolitan University, Oslo, Norway; 5 Department of Finance, Trondheim Municipality, Trondheim, Norway; 6 Department of Health and Welfare, Trondheim Municipality, Trondheim, Norway

**Keywords:** Physical activity, Older adults, Care services, Sedentary behavior, Walking bouts, Daily physical behavior, Accelerometry, Device-measured activity

## Abstract

**Background:**

Physical activity (PA) is imperative for healthy ageing and is a modifiable lifestyle factor. Accurate, clinically meaningful estimates of daily PA among older adults can inform targeted interventions to maintain function and independence. This study describes daily PA in older adults across levels of care as a first step contributing to the limited evidence on potential associations between PA and the use of care services.

**Methods:**

This study used data from the Trondheim 70 + cohort in the population-based Norwegian HUNT Study. In total, 1042 participants aged 70 years or older with valid activity data were included. PA was assessed using two accelerometers over 7 consecutive days and was classified into PA (walking, standing, running, and cycling) and sedentary behavior (sitting and lying). Data on received care services were retrieved from municipal registers and participants were classified into four levels of care: 1) independently living (81.9%), 2) independently living with low-level home care services (6.5%), 3) recipients of home care services (6.0%), and 4) nursing home residents (5.7%). Time spent in the activity types and duration of bouts are presented across levels of care.

**Results:**

Participants mean age was 77.5 years (range: 70.1–105.4, 55% female) and PA was lower with higher age. Across levels of care, significant group differences were found in the total time spent in PA, particularly in walking and standing. Daily PA, duration of active bouts, and number of daily walking bouts were lower for participants receiving higher levels of care. Standing was the dominant type of PA and walking appeared predominantly in short bouts at all care levels.

**Conclusions:**

This is the first population-based study using device-measured PA to describe daily PA across levels of care. The results showed that low-intensity activities constitute the primary component of everyday PA, advocating for placing greater emphasis on the significant role these activities play in maintaining daily PA at older age. Furthermore, the study demonstrated that activity types and bout durations are related to the ability to live independently among older adults. Overall, these findings can contribute to better target interventions to maintain function and independence in older adults.

**Supplementary Information:**

The online version contains supplementary material available at 10.1186/s11556-024-00355-6.

## Background

The proportion of older adults is increasing worldwide, and the number of older adults living with functional disabilities is expected to increase substantially in the coming years [1]. Physical activity (PA), defined as any bodily movement that results in energy expenditure [2], is a modifiable factor well established as an important determinant for maintaining function and independence in daily life [3–5]. Thus, engaging in regular PA and avoiding prolonged sedentary behavior (SB), defined as waking time spent sitting, reclining, or lying down [6], are key strategies to improve prospects of healthy ageing [7, 8]. The updated 2020 recommendations from the World Health Organization (WHO) emphasize the importance of reducing time spent sedentary and highlight that every minute replacing SB with PA of any intensity has health benefits [8]. This has led to a shift from focusing on achieving moderate-to-vigorous PA, towards awareness concerning the complimentary importance of low-intensity PA and its potential role in reducing sedentary time. Everyday such as standing and walking while engaging in household chores or everyday mobility in the local environment, are typically classified as low-intensity PA. These are activities that are essential for many older adults to maintain function and independence in daily life [9]. For most older adults, standing and walking are achievable activities that can counteract the detrimental effects of prolonged SB [9–11]. Low-intensity activities are both feasible and safe for most older adults [12] and have been shown to be associated with lower risk of all-cause mortality [11, 13]. Time spent in low-intensity activities, including standing, are therefore important to recognize and include as part of daily PA in older adults.

Traditional methods quantifying PA and SB, such as questionnaires, are insufficient in capturing the many aspects of PA and are often biased due to recall inaccuracies [14–16]. Furthermore, the ubiquitous nature of low-intensity activities, such as brief bouts of standing and walking as part of everyday life, makes such activities particularly challenging to recall. Given these limitations, activity monitoring using wearable sensors has been established as a more accurate approach for quantifying PA and hence, the preferred method in observational studies [17]. When using wearable sensors, intensity-based cut points are typically applied to categorize time spent in different activity levels, including sedentary, light, and moderate-to-vigorous PA [18, 19]. However, despite adaptations of classical intensity categories to older populations [20–22], the accuracy of these classifications is questionable as older age is associated with a higher metabolic cost of low-intensity daily living activities [18, 20, 23]. Additionally, older adults are a heterogenous group with wide-ranging functional levels, leading to large inter-individual variation in intensity associated with any given activity [22, 24]. It has been shown that using absolute cut points can underestimate the relative intensity of PA in older adults [20, 22, 25]. As a result, their moderate-to-vigorous PA might be misclassified as low-intensity PA. Even more critically, standing and walking at a slow gait speed might be misclassified as SB [22]. Thus, using activity monitoring methods that classify daily PA into activity types and provide accurate data on SB are more appropriate for this population.

The older adult population is characterized by a wide range of functional abilities, spanning from healthy, independently living individuals to frail nursing home residents. Combined with an increasing population of older adults, this provides a rising demand on healthcare services [1, 26]. Care services, whether provided at home or in institutions, refer to personal, social, and medical services that allow people to maintain as much independence as possible [27]. In Norway, care services are provided by municipalities within the publicly financed welfare system, and extensive home care to prevent or delay admission to nursing homes is prioritized. To meet the worldwide challenge of an expanding older population in a sustainable way, there is a need for preventive strategies that reduce and delay the onset of functional disability. In this context, more knowledge regarding daily PA of older adults who receive different levels of care is essential. Nevertheless, the existing evidence on associations between PA and received care services in the older adult population is sparse. A recent study reported an association between PA and non-utilization of long-term care services within a cohort of independently living older adults in Germany [28]. However, these findings were based on self-reported data of both PA and care services. To exploit the potential of PA as a modifiable everyday lifestyle factor, it is essential to obtain accurate measures for clinical use. Specifically, understanding the types of activities in which older adults engage and how the duration of these activities is distributed in daily life, can be used to adapt individual interventions to different functional levels and to different care needs. By integrating accurate daily PA assessments with comprehensive data on care services from a municipality register, we can provide novel insights into the daily PA of older adults. Hence, the aim of this study was to describe daily physical activity patterns in older adults across various levels of care.

## Methods

### *The HUNT4 Trondheim 70* + *cohort*

In the fourth wave of the Trøndelag Health (HUNT4) Study, the catchment area was expanded to include all inhabitants aged 70 and older from a district in the city of Trondheim [29]. Trondheim 70 + followed the same standardized study protocol as used in the HUNT4 70-year-and older (HUNT4 70 +) cohort, previously described in detail [30, 31], and included self-reported questionnaires, clinical examinations, assessments of physical and cognitive performance, and 1-week activity monitoring. Of the invited 5087 individuals, a total of 1745 (34.3%) participated and were assessed by trained health personnel either at a field station (74%), by ambulatory teams in participants’ own homes (11%), or at the nursing home where they lived (15%). The data collection took place from October 2018 until June 2019. Participants provided written informed consent. If they were assessed as unable to consent, consent was provided by their closest proxy. For the current study, all participants in HUNT4 Trondheim 70 + with valid activity data were included (*n* = 1042 (59.7%)). Reasons for missing activity data were: not attached because the participant, nearest proxy, or health personnel did not want to (*n* = 384), technical problems with synchronizing the two sensors in the analyses (*n* = 259), unknown ID due to registration error in data collection (*n* = 16), thigh and back sensors were switched (*n* = 13), or the sensors were removed during the first day (*n* = 31). The current study was approved by the Regional Committees for Medical and Health Research Ethics in Central Norway (157,470).

### Assessment of daily physical activity

Physical activity was assessed using two Axivity AX3 triaxial accelerometers (Axivity, Newcastle, UK) attached directly to the skin on the lower back and the right thigh. Details regarding placement and configuration of the sensors are described elsewhere [32]. The participants were asked to wear the sensors for 7 consecutive days and then return them in a pre-paid envelope. The raw accelerometer data were classified in the activity types walking, standing, sitting, lying, running, and cycling by the Human Activity Recognition 70 + (HAR70 +) model described in detail previously [32, 33].

### Demographics and function

Information on cohabiting status and years of education (grouped based on the educational classification of ISCED11 and NUS 2000 [34]) were obtained from questionnaires. Body mass index (BMI) was calculated from measured height and weight (kg/m^2^). Physical function was assessed with the Short Physical Performance Battery (SPPB), consisting of a hierarchical balance test, a 4-m gait test, and the five-times-sit-to stand test, generating a 0–12 score where higher scores indicate better physical performance [35]. Global cognitive function was assessed with the Montreal Cognitive Assessment (MoCA) [36], a cognitive screening test generating a 0–30 score, where higher scores indicate better cognitive function. Mild cognitive impairment (MCI) and dementia were diagnosed by two scientific and clinical experts for each participant using cognitive and clinical data available from HUNT4 70 + . This process and diagnosis criteria have been described in detail elsewhere [30].

### Care services

Routinely registered data on care services from the municipality of Trondheim were retrieved for the participants at the time of their participation in Trondheim 70 + . Data retrieved were home care services, including nursing care (e.g., medical procedures, administrating medication, assessing health condition), help with personal care (e.g., eating, dressing, toileting, bathing/showering), and assistance with household tasks (e.g., cleaning, shopping, cooking). Time spent in direct contact with recipients was registered and recorded as minutes per visit by the staff. Safety alarm and food service provided by the municipality were also registered. In this study, home care services received by each participant were summed over a week and registered as weekly hours of received care. Based on the types of care services received, we created four groups further referred to as levels of care: 1) independently living receiving no care services (*n* = 853 (81.9%)), 2) independently living with low-level home care services consisting of safety alarm and/or food service only (*n* = 68 (6.5%)), 3) recipients of home care services delivered by health personnel (*n* = 62 (6.0%)), and 4) nursing home residents (*n* = 59 (5.7%)).

The percentage of participants without valid activity data was 32% in both the independently living and low-level home care groups, 65% in the home-care group, and 71% among nursing home residents. Furthermore, participants without valid activity data were older (mean 80.3 years), had lower SPPB (mean 7.4) and MoCA (mean 22.0) scores, and were more likely to be female, compared to the participants with valid activity data.

### Data analysis

The HAR70 + model provided time-stamped continuous activity type classifications in five-second windows [32], and predictions of non-wear time were used to define start- and endpoints of the activity monitoring. Postprocessing and inspection of the individual files with the non-wear and activity type predictions were done with in-house software developed in Python (Python Software Foundation, DE, USA). Based on visual inspections of the raw acceleration data and expert knowledge of human physical behavior, additional filtering of the activity type predictions was applied. First, single five-second windows indicating running or cycling were reassigned to the preceding activity type as it is unlikely that these activities are performed with such short duration. Subsequently, in-bed time was defined based on the activity type predictions for each night, and used to identify the waking time for the analysis of PA. The software used 01:00 am as a predefined starting point, and then identified the end of the previous period with activity that was chosen as start of in-bed time. The end of in-bed time was defined as the first period with activity after 03:00 am. To account for people getting up during the night, the software tested 15-min epochs during the in-bed hours, with < 15% of lying in the epoch defined as not being in-bed time.

To be considered a valid day of activity monitoring, the sensors had to be attached from the beginning to the end of the day. Furthermore, we conducted a visual inspection of the raw data files for those cases in which the sensors had been removed before day 7. This step was conducted to ensure accurate prediction of non-wear time and to inspect predictions that were considered “outliers”. During this procedure, we were able to recognize and exclude files where the sensors had been removed on the first day of recording, as well as cases where the thigh and back sensors had been switched.

PA and SB were averaged across valid days and reported in daily minutes and percentage of time awake. PA contained the activity types walking, standing, running, and cycling. SB contained sitting and lying. Additionally, we calculated walking and active bouts. Active bouts consisted of the activity types walking, standing, running, and cycling, and were categorized based on duration: 10 to < 30 s, 30 to < 60 s, 1 to < 3 min, 3 to < 10 min, 10 to < 60 min and ≥ 60 min. The daily bout averages were computed based on valid days and reported as the mean number of daily bouts. Recognizing that short interruptions of standing are likely during activities such as prolonged walking in free-living environments, a threshold of 15% for other activity types was permissible in bout calculations.

### Statistical analyses

Descriptive statistics were reported as means ± standard deviations (SD) or proportions (n, %) for the categorical variables. Group differences between levels of care were compared group by group using the chi-squared test for discrete variables and the Mann–Whitney U test for continuous variables. PA was visualized in box plots and bar plots. Results are presented by level of care. A multiple linear regression analysis was performed for the association between total PA and care level, adjusted for age, sex, physical performance, and cognitive status. Statistical analyses were performed using Stata Statistical Software version 17 (StataCorp LLC, TX, USA).

## Results

### Participant characteristics

Participants mean age was 77.5 years (range: 70.1–105.4) and 55% were female. In the groups with higher level of care, participants were older and more likely to be female, had lower physical and cognitive performance, and a larger proportion was diagnosed with MCI and dementia (Table 1). In the home care group, the number of weekly hours of care (0.1–22.0 h/week) and types of care varied widely. In total, 87.1% received nursing care (0.1–8.3 h/week). Personal home care was the category with the highest number of weekly hours (0.2–16.0 h/week), while household home care was the category with the smallest number of weekly hours (0.1–2.0 h/week) (Table 1).

**Table 1:** Participant characteristics by level of care

	Independently living (*N* = 853)	Low-level home care (*N* = 68)	Home care (*N* = 62)	Nursing home (*N* = 59)	Group differences
Female, *n (%)*	443 (52)	48 (71)	38 (61)	43 (73)	< .001 ^**a,c**^
Age (years)	75.9 (4.6)	82.7 (6.0)	84.0 (7.1)	88.3 (7.7)	< .001 ^**a,b,c,e,f**^
BMI (kg/m^2^)	26.6 (4.1)	25.9 (4.2)	28.5 (6.9)	26.7 (6.4)	not sig
*missing, n (%)*	*9 (1.1)*	*2 (2.9)*	*4 (6.5)*	*13 (22.0)*	
Gait speed (m/sec)	1.0 (0.2)	0.8 (0.2)	0.6 (0.2)	0.5 (0.2)	< .001 ^**all**^
*missing, n (%)*	*19 (2.2)*	*2 (2.9)*	*4 (6.5)*	*12 (20.3)*	
**SPPB (0–12)**	10.9 (1.8)	8.8 (3.0)	5.9 (3.4)	2.9 (2.7)	< .001 ^**all**^
*missing, n (%)*	*35 (4.1)*	*1 (1.5)*	*2 (3.2)*	*1 (1.7)*	
**MoCA (0–30)**	24.3 (3.3)	23.2 (3.9)	18.4 (4.9)	14.7 (5.0)	< .001 ^**all**^
*missing, n (%)*	*25 (2.9)*	*4 (5.9)*	*10 (16.1)*	*37 (62.7)*	
**Cognitive status,***n (%)*					< .001 ^**all**^
No CI	497 (58.7)	32 (47.8)	9 (14.8)	3 (5.1)	
MCI	311 (36.7)	25 (37.3)	29 (47.5)	3 (5.1)	
Dementia	39 (4.6)	10 (14.9)	23 (37.7)	53 (89.8)	
* missing, n (%)*	*6 (0.7)*	*1 (1.5)*	*1 (1.6)*	*0 (0.0)*	
**Education**					< .001 ^**a,b,c**^
≤ 10 years	71 (8.6)	12 (18.8)	16 (40.0)	12 (40.0)	
11–13 years	313 (38.0)	29 (45.3)	17 (42.5)	10 (33.3)	
≥ 14 years	440 (53.4)	23 (35.9)	7 (17.5)	8 (26.7)	
* missing, n (%)*	*29 (3.4)*	*4 (5.9)*	*22 (35.5)*	*29 (49.2)*	
**Living conditions**					
Living alone	230 (27.9)	49 (77.8)	30 (73.2)		< .001 ^**a,b**^
* missing, n (%)*	*30 (3.5)*	*5 (7.4)*	*21 (33.9)*		
**Home care services**					
Nursing care, *n (%)*			54 (87.1)		
* h/week, mean (SD)*			*1.4 (1.7)*		
Personal home care, *n (%)*			34 (54.8)		
* h/week, mean (SD)*			*2.7 (3.6)*		
Household home care, *n (%)*			24 (38.7)		
* h/week, mean (SD)*			*0.5 (0.5)*		

Group differences were tested group by group using chi-squared test for discrete variables and Mann–Whitney U test for continuous variables. Values are given in mean (SD) for the variables age, BMI, SPPB and MoCA, and proportion (n, %) for the other variables

*BMI* Body mass index, *SPPB* Short Physical Performance Battery, *MoCA* Montreal Cognitive Assessment, *CI* Cognitive impairment, *MCI* Mild cognitive impairment

^a^significant difference (*p* < .001) between Independently living and Low-level home care

^b^significant difference (*p* < .001) between Independently living and Home care

^c^significant difference (*p* < .001) between Independently living and Nursing home

^d^significant difference (*p* < .001) between Low-level home care and Home care

^e^significant difference (*p* < .001) between Low-level home care and Nursing home

^f^significant difference (*p* < .001) between Home care and Nursing home

### Age and sex

**Fig. 1:**
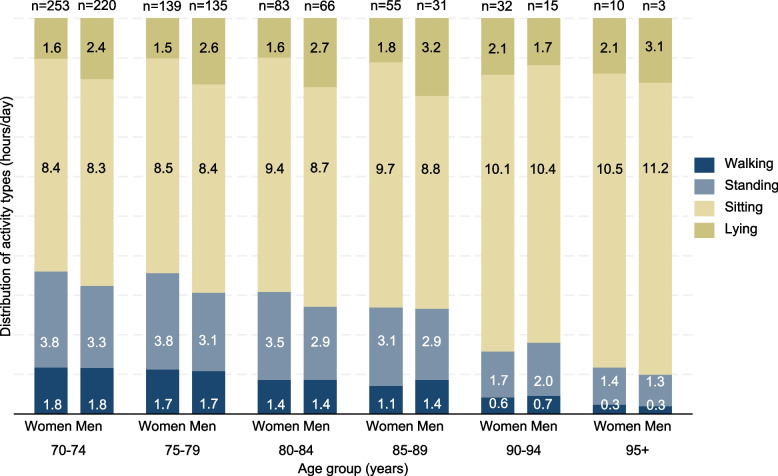
Distribution of activity types by age group and sex. Mean daily hours are showed inside the bars. Number of participants are given at the top

As shown in Fig. 1, PA was lower with higher age. Within age groups, men and women spent nearly equal time walking, but a sex difference was observed in time spent standing up to 85 years. In the age group 85–89 years, men maintained their daily PA whereas women showed a lower level of PA compared to the preceding age groups. From the age of 90 years, significantly lower levels of daily PA were observed for both men and women.

### Daily physical activity across levels of care

**Fig. 2:**
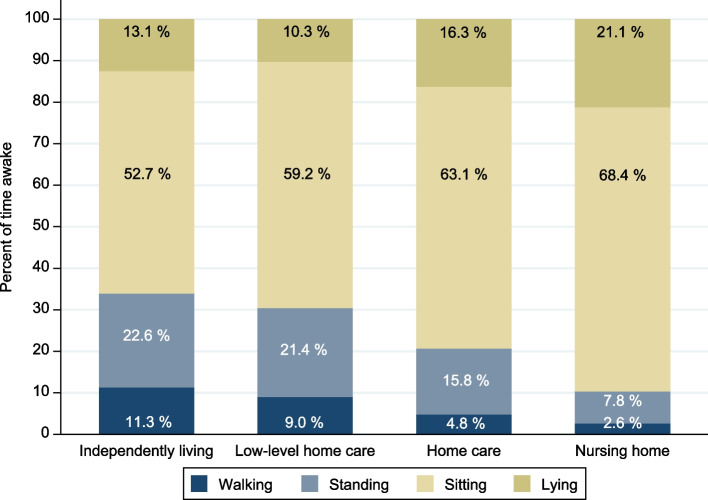
Distribution of activity types in percentage of time awake by level of care

Daily PA was significantly lower with higher levels of care (all *p*´s < 0.001, Table 2). Independently living older adults spent 5.4 h/day in PA, compared to 4.8 h/day in the low-level home care group, 3.3 h/day in the home care group, and 1.6 h/day in the nursing home group. Daily time spent walking was 1.8 h/day for independently living, 1.4 h/day for recipients of low-level home care, 0.8 h/day for recipients of home care, and 0.4 h/day for those living in a nursing home (all *p*´s < 0.001, Table 2 and Fig. 2). A regression analysis, adjusted for age, sex, physical performance, and cognitive status, showed that lower daily PA with higher level of care was largely explained by higher age, more women, lower physical performance, and lower cognitive function in the groups with a higher care level compared to the independently living group. The adjusted model estimates (95% CI) showed that the difference in total PA (min) were -9.5 (-33.8 to 14.8 (*p* = 0.444)) for the low-level home care group, -40.5 (-69.1 to -12.0 (*p* < 0.005)) for the home care group, and -97.8 (-133.9 to -61.7 (*p* < 0.001)) for the nursing home residents, compared to the independently living group. Full model results are shown in Table 3 (see Additional file 1).

**Table 2:** Distribution of daily physical activity by level of care

	Independently living (*N* = 853)	Low-level home care (*N* = 68)	Home care (*N* = 62)	Nursing home (*N* = 59)	Group differences
	Mean (SD)	Mean (SD)	Mean (SD)	Mean (SD)	
**Total PA (%)**	**34.1 (10.2)**	**30.5 (9.9)**	**20.7 (12.7)**	**10.4 (8.1)**	< .001 ^**all**^
Walking (%)	11.3 (4.2)	9.0 (3.8)	4.8 (3.7)	2.6 (2.7)	< .001 ^**all**^
Standing (%)	22.6 (7.7)	21.4 (7.6)	15.8 (10.0)	7.8 (6.1)	< .001 ^**b,c,d,e,f**^
Running (%)	0.0 (0.1)	0.0 (0.0)	0.0 (0.0)	0.0 (0.0)	< .001 ^**a,b,c**^
Cycling (%)	0.2 (0.5)	0.1 (0.2)	0.02 (0.1)	0.1 (0.3)	< .001 ^**b,c,d,e**^
**Total SB (%)**	**65.7 (10.4)**	**69.5 (9.9)**	**79.3 (12.7)**	**89.6 (8.1)**	< .001 ^**all**^
Sitting (%)	52.7 (13.6)	59.2 (12.2)	63.1 (18.6)	68.4 (16.0)	< .001 ^**a,b,c,e**^
Lying (%)	13.1 (11.9)	10.3 (10.4)	16.3 (17.6)	21.1 (14.0)	< .001 ^**c,e**^
**Total PA (min)**	**322 (98)**	**286 (93)**	**197 (122)**	**95 (77)**	< .001 ^**all**^
Walking (min)	107 (40)	84 (37)	46 (34)	23 (25)	< .001 ^**all**^
Standing (min)	213 (75)	201 (71)	152 (97)	71 (59)	< .001 ^**b,c,d,e,f**^
Running (min)	0 (1)	0 (0)	0 (0)	0 (0)	< .001 ^**a,b,c**^
Cycling (min)	1.9 (5.3)	0.8 (1.5)	0.2 (0.8)	0.6 (2.4)	< .001 ^**b,c,d,e**^
**Total SB (min)**	**621 (113)**	**655 (119)**	**761 (164)**	**786 (132)**	< .001 ^**all**^
Sitting (min)	499 (135)	559 (131)	603 (184)	598 (153)	< .001 ^**a,b,c,e**^
Lying (min)	122 (108)	96 (97)	158 (167)	188 (128)	< .001 ^**c,e**^
**Total AB (nr)**	**51.3 (18.1)**	**46.0 (15.0)**	**42.8 (20.3)**	**35.1 (23.6)**	< .001 ^**a,b,c,e**^
10–30 s (nr)	9.2 (9.7)	7.3 (8.0)	6.5 (7.5)	7.0 (12.5)	< .001 ^**b,c**^
30–60 s (nr)	6.9 (3.6)	6.0 (2.9)	5.9 (3.9)	5.0 (3.9)	< .001 ^**b,c**^
1–3 min (nr)	13.0 (5.1)	12.1 (5.4)	12.9 (7.3)	12.6 (7.6)	not sig
3–10 min (nr)	12.6 (4.2)	11.9 (4.2)	12.0 (6.4)	8.7 (6.6)	< .001 ^**c,e,f**^
10–60 min (nr)	8.9 (3.1)	8.3 (2.9)	5.3 (4.2)	1.7 (2.5)	< .001 ^**b,c,d,e,f**^
≥ 60 min (nr)	0.7 (0.6)	0.5 (0.5)	0.2 (0.4)	0.0 (0.1)	< .001 ^**b,c**^
**Total WB (nr)**	**90.6 (38.5)**	**70.9 (33.3)**	**43.3 (36.7)**	**23.2 (27.5)**	< .001 ^**all**^
10–30 s (nr)	42.3 (17.3)	34.4 (15.9)	22.5 (18.7)	9.1 (11.1)	< .001 ^**all**^
30–60 s (nr)	29.7 (13.8)	23.2 (12.4)	14.1 (12.8)	8.0 (9.6)	< .001 ^**all**^
1–3 min (nr)	15.0 (8.8)	10.6 (6.0)	5.7 (5.3)	5.8 (7.1)	< .001 ^**a,b,c,d,e**^
3–10 min (nr)	2.6 (1.8)	1.9 (1.5)	0.9 (1.3)	0.4 (0.7)	< .001 ^**all**^
10–60 min (nr)	1.1 (1.0)	0.8 (0.8)	0.2 (0.3)	0.0 (0.1)	< .001 ^**b,c,d,e**^
≥ 60 min (nr)	0.0 (0.1)	0.0 (0.1)	0.0 (0.0)	0.0 (0.0)	< .001 ^**b,c**^
**Valid days**	5.8 (1.0)	5.7 (1.4)	5.8 (1.3)	6.0 (0.7)	not sig

The activity types, total PA and total SB are reported in daily minutes and percentage of time awake. Number of active bouts (AB) and walking bouts (WB) are presented in categories based on duration. Group differences were tested group by group using chi-squared test for discrete variables and Mann–Whitney U test for continuous variables

^a^significant difference (*p* < .001) between Independently living and Low-level home care

^b^significant difference (*p* < .001) between Independently living and Home care

^c^significant difference (*p *< .001) between Independently living and Nursing home

^d^significant difference (*p* < .001) between Low-level home care and Home care

^e^significant difference (*p* < .001) between Low-level home care and Nursing home

^f^significant difference (*p* < .001) between Home care and Nursing home

*PA* Physical activity, *SB* Sedentary behavior, *AB* Active bouts, *WB* Walking bouts

### Physical activity patterns

**Fig. 3:**
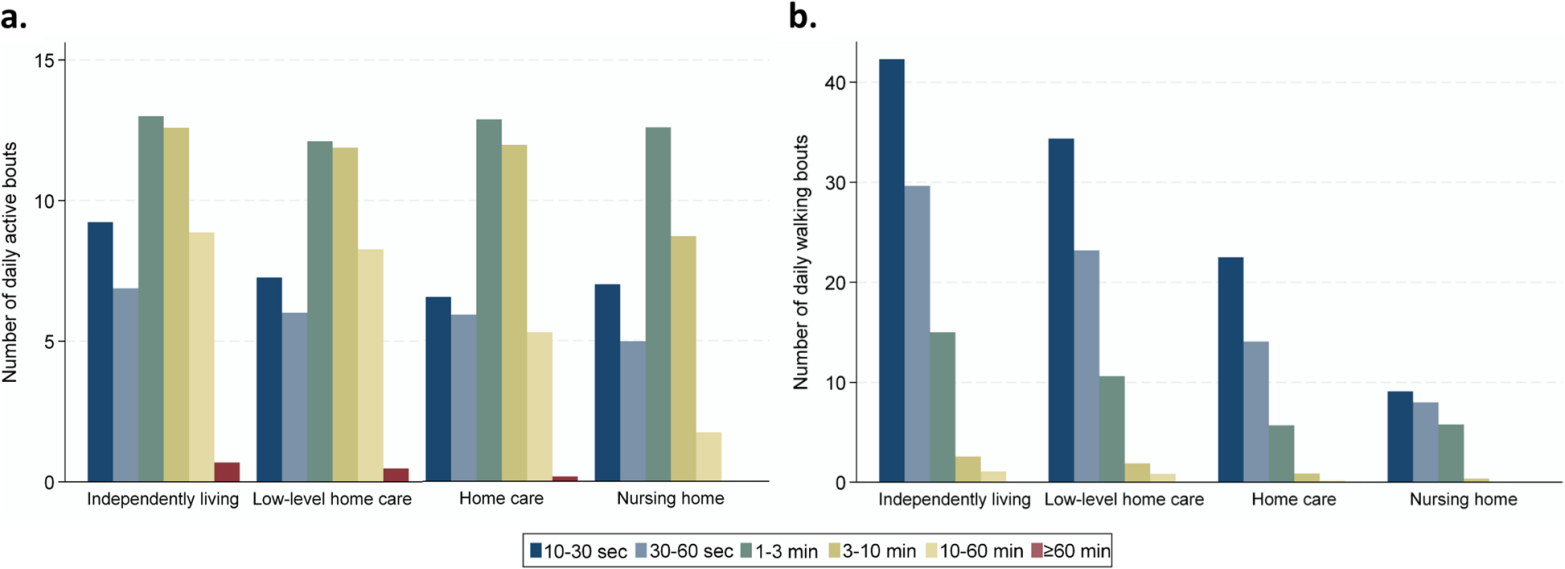
Number of daily active bouts (**a**) and number of daily walking bouts (**b**) in categories of bout duration by level of care. Walking bouts contain walking only, while active bouts contain the activity types walking, standing, running, and cycling. Note that the scale of the y-axis is different in the two plots

Number of walking and active bouts were also lower with higher level of care (Table 2). The distribution of walking bouts showed the same pattern for all levels of care. The highest number was achieved in the shortest duration category (10-30s) and thereafter steadily fewer bouts in the longer duration categories (Fig. 3b). For active bouts (including the activities walking, standing, running, and cycling), most bouts had a duration between 1 and 10 min at all care levels (Fig. 3a). The daily number of active bouts with longer durations (10–60 and ≥ 60 min) were substantially lower at higher care level. The independently living and low-level home care groups had 8.9 and 8.3 daily active bouts lasting between 10 and 60 min, whereas those receiving home care and nursing home residents had 5.3 and 1.7 bouts, respectively (Table 2). However, there was large variation between individuals, with active bouts ≥ 60 min ranging from 0 to 3.8 per day. Among the independently living older adults, 17.7% had no active bouts above 60 min during the activity monitoring period. The corresponding values for the low-level home care, home care and nursing home groups, were 29.4%, 69.4% and 94.9%, respectively. In total, 25.9% of the independently living individuals, 14.7% of the recipients of low-level home care, 3.8% of the recipients of home care, and none of the nursing home residents had on average at least one daily active bout above 60 min.

## Discussion

This study described daily physical activity patterns, focusing on activity types and the duration of active bouts, in older adults across various levels of care. The results in this study revealed that low-intensity activities constitute the primary component of everyday PA in older adults. The total time spent in PA, particularly in walking and standing, were lower at higher care levels compared to independently living older adults. With higher levels of care, daily PA, duration of active bouts, and number of daily walking bouts were lower. Furthermore, standing was the dominant type of PA and walking appeared predominantly in short bouts at all care levels.

### Device-measured physical activity

The current study showed that standing was the primary type of PA in older adults. While previous studies mainly focused on intensity or SB, few have classified activity types in older adults using wearable sensors. Rosenberg et al. (2020) reported an average standing time of 3.9 h daily among 1039 independently living older adults [37], which is comparable to our study where independently living older adults spent an average of 3.6 h standing. Previous studies that described time spent walking, reported averages ranging from 1.3 to 1.8 h daily for independently living older adults [37–40]. In comparison, in this study independently living older adults spent an average of 1.8 h walking daily. Classification into activity types does not account for the intensity of activities. Thus, evaluating to what extent recommendations for PA are met within the sample was not assessed in this study. However, the revised WHO recommendations emphasize the essential role of low-intensity PA for reducing sedentary time and indicate that every minute replacing SB with PA has health benefits [8]. This implies that a shift from focusing on achieving moderate-to-vigorous PA towards awareness concerning the importance of low-intensity PA is imperative. Classifying physical behavior into activity types can provide accurate estimates of both low- and higher-intensity PA, which can be used to further elaborate recommendations for daily PA in older adults.

A recent review by Chastin et al. (2021) pointed to insufficient evidence for effective interventions in reducing SB among older adults and recommended future studies to use methods capable of identifying breaks in SB and distinguishing between various postures [41]. The current study introduces a new perspective on how older adults accumulate PA by examining bout durations. By not capturing short bouts of standing and walking, earlier studies likely have underestimated PA, especially since self-reporting low-intensity activities is challenging due to the ubiquitous nature of daily living PA [14–16]. Additionally, intensity-based cut-point approaches are limited in capturing short bouts, as they analyze data in longer epochs, typically 15–60 s, in contrast to the 5-s windows used in our approach [32]. Describing daily PA based on activity types and bout durations could be valuable in guiding future research on interventions aimed at reducing SB by providing more accurate estimates that capture even short bouts of PA.

The current study showed that there are substantial age and sex differences in daily PA in older adults. In general, PA was lower with higher age for both men and women, but while men and women spent nearly equal time walking, women spent more time standing than men. Previous studies have shown that men are generally more active than women [42]. However, this might be affected by how activity was classified. Our findings indicate that women spend more time upright than men, which contributes important information for understanding daily PA among older adults.

### Physical activity patterns across levels of care

Not surprisingly, the participants in the groups with higher levels of care were older, had worse cognitive and physical function, and were more often female than the independently living participants. The regression analysis showed that the differences between care levels for time spent in daily PA were largely explained by these factors (Additional file 1). There was no significant difference in daily PA between the low-level home care group and those living independently at home. The differences observed in Table 2 were likely related to differences between men and women. Women spend, on average, 35 min more time per day on PA than men (Additional file 1). Therefore, the differences in daily PA across care levels are likely influenced by the higher proportion of women at higher care levels. Our findings indicate that studies aiming to describe the association between daily PA and care level should consider these factors. As a first step in contributing to the limited evidence on daily PA related to the use of care services, the current study provides clinically relevant measures of PA in older adults. These measures can be further used to investigate potential associations between daily PA and care services.

Investigating not only the total volume of PA but also bout durations provides valuable information about daily activity in older adults, as we observed that daily PA occurs over a wide range of durations. Walking bouts and active bouts, the latter also containing standing, should be seen in context to each other as both provide insight into patterns of accumulated PA in older adults. Our results showed that walking is distributed over many daily bouts and appears predominantly in short bouts lasting less than a minute, consistent with previous studies [43–45]. The distribution of walking bouts showed a consistent pattern with gradually fewer bouts with longer lengths, although number of bouts were significantly lower with higher level of care.

Extracting contextual information from free-living PA data is challenging. Following Del Din et al. (2016), we assume that most walking bouts up to one minute likely represent walking indoors, while longer durations are likely to correspond to walking outdoors [45]. However, even during outdoor activities, prolonged walking often involves brief stationary periods, such as when waiting for traffic, navigating through crowded areas, or taking a moment to catch one’s breath while enjoying the scenery. Likewise, during household chores and personal care activities at home, brief walking bouts interspersed with varying durations of standing are the primary type of activity. In any context, the way interruptions are considered during analysis of activity data will have a significant impact on defining walking bouts during free-living activity in older adults [46, 47]. This study showed that episodes of walking are significantly interspersed with episodes of standing in older adults. The lengthened durations of active bouts, and a considerably lower total number of active bouts compared to walking, reveals that periods of standing are a dominant component of upright activities in older adults’ daily life. Furthermore, this study showed that prolonged uninterrupted walking does not occur often. Independently living older adults averaged only about one daily walking bout lasting longer than 10 min, indicating that most older adults either do not engage in prolonged walks or break them up in shorter walking periods.

One of the most prominent differences between care levels was the lower number of walking bouts with higher level of care, which was consistent across all bout duration categories. A larger number of bouts implies that individuals have more transitions between activity types, reflecting larger variation in daily PA, which previously was observed in individuals with better health status [48, 49]. The observed differences in daily PA variation between care levels might reflect individuals’ ability to live independently. This information could be clinically relevant in identifying individuals at risk of functional decline at an early stage and targeting interventions to help them regain or maintain their independence. This study showed that individuals living independently have higher variation in their daily PA than those receiving care services. This suggests that older adults unable to maintain variation in PA may be at risk of requiring care services. The potential of daily PA variation as indicator of functional decline together with targeted interventions focusing on increasing variation by breaking prolonged SB, should be further investigated in longitudinal studies.

Another interesting finding regarding indicators related to functional decline is the observed difference between the low-level home care group, that receives only safety alarm and/or food service, and the home care group. The first group did not differ substantially from independently living older adults, neither in total PA volume nor bouts. Although active bouts with durations up to 10 min were maintained in the home care group compared to the low-level home care group, a significantly lower number of active bouts lasting longer than 10 min was observed among those receiving home care. This difference may indicate that those receiving a higher level of care have stopped going outdoors, and instead engage in everyday indoor activities of shorter durations. Individuals classified in the low-level home care group may be well-suited for effectively implementing preventive measures, especially within a municipality-based care system such as in Norway. When assigned a safety alarm or receiving food service, the care needs of these individuals have been identified by care workers, and individuals are registered in the community services. Implementing interventions at this stage aimed at preserving independence, such as increasing PA, may hold great potential for reducing or delaying the need for additional care services. This is crucial for maintaining independence and the quality of life for the individuals and their close ones. According to the findings in this study, prioritizing activity type-specific measures, especially those aimed at maintaining variation and active bout durations at this stage, may hold potential to reduce or delay the need for additional care services.

To the best of our knowledge, this is the first population-based study using device-measured activity to describe daily PA across levels of care. Steinbeisser et al. (2022) have recently reported an association between PA and non-utilization of long-term care services from a large population study in older adults [28]. However, data of both PA and care services were self-reported and compared PA for older adults with and without care services. Care services are often multifaceted and vary considerable in type and amount of care given. Thus, the current study contributes with additional valuable information for understanding daily activity among older adult across levels of care.

### Strengths and limitations

One of the main strengths of this study is the use of device-measured activity data, classified into activity types with high accuracy using five-second windows. The activity data was analyzed using a validated machine learning model for fit-to-frail older adults, including those using walking aids [32]. This approach enables the recognition of everyday activities typical for the target population. Additionally, the current study introduces an analysis capable of investigating bout durations, addressing a previously identified research gap [50]. Another strength is the breadth of the sample concerning age and cognitive and physical function. Finally, the comprehensive data on care services retrieved from the municipal registers is a main strength. However, there are some limitations to this work as well. With 40% of the sample missing activity data, and a higher proportion at higher levels of care, the resulting sample likely represents a healthier subset compared to the general population of older adults. Additionally, we do not have access to care services provided by others than the municipality, such as family, friends, or private care providers. This implies that some participants may have received informal care in addition to the care services provided by the municipality. Another important note is that causality in the association between daily PA patterns and level of care cannot be determined due to the cross-sectional data of the current study. Finally, there may be a limitation in classifying activity data without accounting for the intensity of the activities. Nevertheless, accurately classifying activity types represents an essential initial step for estimating relevant measures for clinical use of daily PA, so that further developments in analysis strategies can involve classification of intensity, such as walking speed.

## Conclusions

This study offers novel insights into daily PA patterns and addresses a current evidence gap concerning the free-living PA of older adults across levels of care. The current study shows that low-intensity activities constitute the primary component of everyday PA and advocates for placing larger emphasis on the significant role these activities play in maintaining daily PA at older age. As daily PA, duration of active bouts, and the number of daily walking bouts were lower with higher level of care, our findings suggest that activity types and bout durations are related to the ability to live independently. These measures may be clinically relevant for assessing PA in older adults. Accurate estimates of daily PA among older adults can be used for evaluation of physical behavior and targeted interventions to maintain function and independence in older adults.

### Supplementary Information

Additional file 1: Table 3. Regression results for the association between total PA (minutes) and care level.

## Data Availability

Data is not publicly available. Data may be obtained from the HUNT database (https://www.ntnu.edu/hunt). Data on care services may be obtained from the Norwegian Registry for Primary Health Care (https://helsedata.no/en/forvaltere/norwegian-directorate-of-health/norwegian-registry-for-primary-health-care-kpr/).

## Abbreviations

PA: Physical activity
SB: Sedentary behavior
WHO: World Health Organization
BMI: Body mass index
SPPB: Short Physical Performance Battery
MoCA: Montreal Cognitive Assessment
MCI: Mild cognitive impairment
