# Supplementary material for: Daily physical activity in older adults across levels of care: the HUNT Trondheim 70 + study
Source: Eur Rev Aging Phys Act. 2024 Jul 17;21:20. doi: 10.1186/s11556-024-00355-6 (PMC11253329; doi:10.1186/s11556-024-00355-6)
Supplement: Supplementary file 1 — Additional file 1: Table 3. Regression results for the association between total PA (minutes) and care level. [file 11556_2024_355_MOESM1_ESM.docx]

### Additional file 1

**Table 3.** Regression results for the association between total PA (minutes) and care level.

|  | **Unadjusted model** | | **Adjusted model*** | |
| --- | --- | --- | --- | --- |
|  | Coefficient (95% CI) | p | Coefficient (95% CI) | p |
| **Care level** |  |  |  |  |
| Independently living | Ref. |  | Ref. |  |
| Low-level home care | -36.1 (-60.4 to -11.8) | .004 | -9.5 (-33.8 to 14.8) | .444 |
| Home care | -124.9 (-150.3 to -99.4) | <.001 | -40.5 (-69.1 to -12.0) | .005 |
| Nursing home | -227.5 (-253.5 to 201.5) | <.001 | -97.8 (-133.9 to -61.7) | <.001 |
| **Sex** |  |  |  |  |
| Female | . | . | Ref. |  |
| Male | . | . | -34.8 (-46.2 to -23.3) | <.001 |
| **Age** | . | . | -0.9 (-2.0 to 0.2) | .139 |
| **SPPB (0-12)** | . | . | 14.8 (11.8 to 17.8) | <.001 |
| **Cognitive status** | . | . |  |  |
| No cognitive impairment | . | . | Ref. |  |
| Mild cognitive impairment | . | . | -13.4 (-25.8 to -0.9) | .035 |
| Dementia | . | . | -14.5 (-38.6 to 9.5) | .237 |
| **Constant** | 322.2 |  | 251.9 |  |

* Model adjusted for age, sex, physical function (SPPB), and cognitive status. The number of participants for the adjusted model was n=997.

Ref.: The independently living group, female and no cognitive impairment were selected as reference.

PA: Physical activity, CI: Confidence interval, SPPB: Short Physical Performance Battery
